# Two Metagenomes from Late Pleistocene Northeast Siberian Permafrost

**DOI:** 10.1128/genomeA.01380-14

**Published:** 2015-01-02

**Authors:** Kirill Krivushin, Fyodor Kondrashov, Lyubov Shmakova, Mariya Tutukina, Lada Petrovskaya, Elizaveta Rivkina

**Affiliations:** a Institute of Physicochemical and Biological Problems in Soil Science, Russian Academy of Sciences, Pushchino, Russia; b Institució Catalana de Recerca i Estudis Avançats (ICREA), Centre for Genomic Regulation (CRG), and Universitat Pompeu Fabra (UPF), Barcelona, Spain; c Institute of Cell Biophysics, Russian Academy of Sciences, Pushchino, Russia; d Shemyakin and Ovchinnikov Institute of Bioorganic Chemistry, Russian Academy of Sciences, Moscow, Russia

## Abstract

The present study reports metagenomic shotgun sequencing of microbial communities of two ancient permafrost horizons of the Russian Arctic. Results demonstrate a significant difference in microbial community structure of the analyzed samples in general and microorganisms of the methane cycle in particular.

## GENOME ANNOUNCEMENT

Microorganisms in Arctic permafrost retain viability for thousands and millions of years (1). The deposits that did not thaw during the Holocene optimum in northeast Siberia arouse particular interest. They are considered a potential pool of buried organic carbon and greenhouse gases (2, 3).

This study reports metagenomic shotgun sequencing of the two permafrost samples of different origin and very similar age.

Permafrost sediments were sampled in the Kolyma-Indigirka Lowland in northeast Siberia (69°299 N, 156°599E) in the 2007 summer field season, as described elsewhere (4). Sample IC4 corresponds to the permafrost sediment of a lake originating from the Panteleikha River floodplain: DH-2007/4; depth, −22.5 m; 1 to 2 mmol CH_4_/kg; 30,696 ± 394 years (5). IC8 was sampled from the late Pleistocene Ice Complex on the Omolon River: DH-2007/2; depth, 6 m; no methane detected. The age of the second sample was assessed as 32,000 years, due to the origin of the earlier described outcrop (6).

Eight biological replicates of 200 to 300 mg each were used for the total DNA extraction with the Power Soil DNA extraction kit (MoBIO, USA). Due to low yield, DNA was concentrated applying the Genomic DNA clean and concentrator kit (Zymo Research, USA). Metagenome sequencing was performed at the CRG ultra-sequencing facility (Centre for Genomic Regulation, Spain) using the TruSeq SBS kit version 3 and the Illumina HiSeq 2000 machine. A 2 × 100 cycle sequencing protocol was used.

A total of 2,000,000 raw reads per sample were submitted to the MG-RAST server (7) for taxonomic annotation against M5NR (8) databases at default parameters. Previously processed mate pair reads were treated with cutadapt (9) to remove adapter contamination and merged with a fastq-join utility (10).

Comparison of taxonomic distribution of both samples demonstrated that bacteria prevail in the IC8 sample, while archaea are more abundant in IC4. More detailed analysis showed that Mycobacterium, Bradyrhizobium, Rhodopseudomonas, and Hyphomicrobium abound in the first metagenome, whereas Conexibacter, Streptomyces, *Nakardiodes*, and Frankia are more widespread in the second metagenome. Thus, the predominance of some genera might be considered distinctive features of the metagenomes IC4 and IC8, respectively.

As presence and absence of biogenic methane is a distinctive feature of samples IC4 and IC8, respectively, the distribution of methanogens in the samples is an interesting result. The methanogenic archaea contribute up to 0.5% to the microbial communities. *Methanosarcinaceae* and *Methanobacteriaceae* are the most widely distributed families detected in both metagenomes. Meanwhile, methanogenic archaea are more abundant in sample IC4 than in IC8. A similar trend is found with methylotrophic bacteria. Thus, prevalence of methane-cycling microorganisms is another distinctive feature of the sample IC4.

In this study, we found a significant difference in the taxonomic structure of two permafrost samples of similar age, presumably related to the different geneses of these deposits. Further analysis is planned to reveal factors causing the discrepancy.

### Nucleotide sequence accession numbers.

The nucleotide sequences from this metagenomic project were deposited at DDBJ/EMBL/GenBank under the accession numbers SRX763249 and SRX751044.
